# A Pan-Cancer Analysis of the Oncogenic Role of Twinfilin Actin Binding Protein 1 in Human Tumors

**DOI:** 10.3389/fonc.2021.692136

**Published:** 2021-05-25

**Authors:** Gengwei Huo, Yali Wang, Jinliang Chen, Ying Song, Cuicui Zhang, Hua Guo, Ran Zuo, Fuyi Zhu, Jinfang Cui, Weidong Chen, Wenming Chen, Peng Chen

**Affiliations:** ^1^ Department of Thoracic Oncology, Lung Cancer Diagnosis and Treatment Center, Tianjin Medical University Cancer Institute and Hospital, National Clinical Research Center for Cancer, Key Laboratory of Cancer Prevention and Therapy of Tianjin, Tianjin’s Clinical Research Center for Cancer, Tianjin, China; ^2^ Department of Oncology, Jining No.1 People’s Hospital, Jining, China; ^3^ Department of Pharmacy, Jining No.1 People’s Hospital, Jining, China; ^4^ Department of Tumor Cell Biology, Tianjin Medical University Cancer Institute and Hospital, Tianjin, China

**Keywords:** TWF1, cancer, prognosis, phosphorylation, survival

## Abstract

**Background:**

Understanding common and unique mechanisms driving oncogenic processes in human tumors is indispensable to develop efficient therapies. Recent studies have proposed Twinfilin Actin Binding Protein 1 (TWF1) as a putative driver gene in lung cancer, pancreatic cancer and breast cancer, however a systematic pan-cancer analysis has not been carried out.

**Methods:**

Here, we set out to explore the role of TWF1 in 33 tumor types using TCGA (The Cancer Genome Atlas), GEO (Gene Expression Omnibus) dataset, Human Protein Atlas (HPA), and several bioinformatic tools.

**Results:**

As part of our analysis, we have assessed *TWF1* expression across tumors. We found that over-expression of *TWF1* generally predicted poor OS for patients with tumors with high *TWF1* expression, such as mesothelioma, lung adenocarcinoma, cervical cancer and pancreatic adenocarcinoma. We also assessed the mutation burden of *TWF1* in cancer and the *TWF1*-associated survival of cancer patients, compared the phosphorylation of TWF1 between normal and primary tumor tissues and explored putative functional mechanisms in TWF1-mediated oncogenesis.

**Conclusions:**

Our pan-cancer analysis provides a comprehensive overview of the oncogenic roles of TWF1 in multiple human cancers.

## Highlights

This comprehensive pan-cancer analysis provides a very thorough overview of the oncogenic roles of TWF1 in multiple human cancers.

1. A first pan-cancer analysis of TWF1.
2. TWF1 is differentially associated with the prognosis of different tumor cases.
3. The link between TWF1 and endothelial cell, neutrophil or cancer-associated fibroblast infiltration.
4. Focal adhesion and vesicle transportation associated issue are involved in the oncogenic role of TWF1.

## Introduction

Identification and characterization of novel pan-cancer genes are crucial to better understand the extremely complex process of tumorigenesis. Publicly funded cancer genomics databases and repositories, such as TCGA (The Cancer Genome Atlas) and GEO (Gene Expression Omnibus), a large number of tumor related functional genomics datasets are available from multiple cancers for more detailed downstream pan-cancer analysis (1–3).

TWF1 (Twinfilin Actin Binding Protein 1) protein, also known as PTK9 or A6, was originally identified in *Saccharomyces cerevisiae* through its sequence homology to ADF/cofilin proteins (4, 5), and it was found to be conserved from yeast to mammals. The protein is composed of two actin-depolymerizing factor (ADF)-homology domains and TWF1 is belonging to the actin depolymerizing factor homology family (6). TWF1 is primarily a cytosolic protein that binds to and sequesters large amounts of actin monomer, thus affecting the assembly of the actin cytoskeleton (7). Mechanistically, TWF1 directly bind to actin filaments, promoting degradation and turnover of actin structures (8, 9). As a consequence, TWF1 is involved in the regulation of diverse morphological and motile processes (10, 11), and has been implicated in cell motility, sensitivity to drugs and cancer progression (7, 12, 13). The previously described role of TWF1 in the regulation of cell cycle and its inhibitory role in tumor growth and metastasis raise the possibility that TWF1 plays a critical role in the development and progression of cancer (14). However, previous studies limited the evaluation of TWF1 to a few cancer types, and its role remain elusive in other tumor types.

To explore expression profile of TWF1 across various tumor types in a pan-cancer analysis, we used dataset available *via* TCGA Project and GEO databases. In addition to expression profile comparison of TWF1 across tumor types, we also considered survival status, genetic alteration, protein phosphorylation and relevant cellular pathways. This comprehensive analysis reveals potential molecular mechanism of TWF1 in the pathogenesis and clinical prognosis of multiple human cancers.

## Materials and Methods

### Gene Expression Analysis

TIMER2 (tumor immune estimation resource, version 2, http://timer.cistrome.org/) was used for the analysis the expression profiling of *TWF1* between tumor types and adjacent normal tissues. In cases, where tumors are typically without or only with limited normal tissue [e.g., TCGA-DLBC (Lymphoid neoplasm diffuse large B-cell lymphoma), TCGA-LGG (Brain lower grade glioma), etc.], we used the GEPIA2 (Gene Expression Profiling Interactive Analysis, version 2) tool (http://gepia2.cancer-pku.cn/#analysis) to acquire box plots of the GTEx (Genotype-Tissue Expression) database, under the settings of *P*-value cutoff = 0.01, log2FC (fold change) cutoff =1, and “Match TCGA normal and GTEx data”. HEPIA2 tool was used to analyze the *TWF1* expression in different pathological stages of all TCGA cancers. The log2 [TPM (Transcripts per million) +1] transformed expression data were applied for the box or violin plots.

We used UALCAN tool (http://ualcan.path.uab.edu/analysis-prot.html) to analyze cancer Omics data, and conduct protein expression analysis of the CPTAC (Clinical proteomic tumor analysis consortium) dataset (15). Expression level of the total protein or phosphoprotein of TWF1 has been compared between primary and normal tissues, respectively.

### Immunohistochemistry (IHC) Staining

To evaluate differences in TWF1 expression at the protein level, IHC images of TWF1 protein expression in normal tissues and six tumors tissues, including LUAD (Lung Adenocarcinoma), BRCA (Breast invasive carcinoma), OV (Ovarian Serous Cystadenocarcinoma), LIHC (Liver hepatocellular carcinoma), TGCT (Testicular germ cell tumors), and THCA (Thyroid carcinoma) were downloaded from the HPA (Human Protein Atlas) (http://www.proteinatlas.org/) and analyzed.

### Survival Prognosis Analysis

We used GEPIA2 to obtain the OS (Overall Survival) and DFS (Disease-Free Survival) significance map data and survival plots of TWF1 across all TCGA tumors. Cutoff-high (50%) and cutoff-low (50%) values were used as the expression thresholds for splitting the high-expression and low-expression cohorts (16). The log-rank test was used in the hypothesis testing.

### Genetic Alteration Analysis

We used cBioPortal tool (https://www.cbioportal.org/) to collect the data of alteration frequency, mutation type, mutated site information, CNA (Copy number alteration) and 3D (Three-dimensional) structure of the protein structure across all TCGA tumors. Survival data, including OS and DFS were compared for all the TCGA cancer types, with or without *TWF1* genetic alteration.

### Immune Infiltration Analysis

TIMER2 tool was used to analyze the relationship between *TWF1* expression and immune infiltrates across all TCGA tumors. Cancer-associated fibroblast, neutrophil and endothelial cell were selected for detailed analysis. The TIMER, TIDE, CIBERSORT, CIBERSORT-ABS, QUANTISEQ, XCELL, MCPCOUNTER and EPIC algorithms were applied for estimations.

### TWF1-Related Gene Enrichment Analysis

We used STRING website (https://string-db.org/) for the subsequent analysis of protein-protein interaction network. The main parameters were: minimum required interaction score [“Low confidence (0.150)”], meaning of network edges (“evidence”), max number of interactors to show (“no more than 50 interactors” in 1st shell) and active interaction sources (“experiments”).

GEPIA2 was used to obtain the top 100 *TWF1*-correlated genes based on the datasets of all TCGA tumor and normal tissues. Then we conducted a pairwise gene-gene Pearson correlation analysis between *TWF1* and the selected genes. The *P*-values and the correlation coefficient (R) were calculated and are indicated in the corresponding figure panels. Heatmap representation of the expression profile for the selected genes contains the partial correlation (cor) and *P*-value in the purity-adjusted Spearman’s rank correlation test.

We merged and filtered the two sets of data to perform KEGG (Kyoto encyclopedia of genes and genomes) pathway analysis. The enriched pathways were visualized with the “tidyr” and “ggplot2” R packages. The R language software [R-3.6.3, 64-bit] (https://www.r-project.org/) was used in this analysis. Two-tailed *P*<0.05 was considered to be statistically significant (17).

## Results

### Gene Expression Analysis Data

In this study, we aimed to provide a comprehensive analysis regarding the putative oncogenic role of human TWF1 (NM_001242397.2 for mRNA or NP_001229326.1 for protein, **Figure S1A**). In our initial analysis, we focused on to explore how conserved TWF1 is across different species. As shown in **Figure S1B**, the TWF1 protein structure is conserved among different species (e.g., H. sapiens, D. melanogaster, K.lactis, etc.) and commonly consists of the ADF_gelsolin (cl15697) domain. The phylogenetic tree data (**Figure S2**) confirmed the evolutionary relationship of the TWF1 protein among the studied species. Altogether, the high level conservation indicate that TWF1 may play important role in fundamental biological processes.

Next, we analyzed the expression pattern of *TWF1* in different cell lines and non- tumor tissues. As shown in **Figure S3A**, we collected data from HPA, GTEx, and FANTOM5 (Function annotation of the mammalian genome 5) dataset. This comparison revealed high expression of *TWF1* in the esophagus, ductus deferens and seminal vesicle, but *TWF1* was ubiquitously expressed in essentially all tissues (all consensus normalized expression values >1), and its mRNA expression showed overall low tissue specificity. When analyzing HPA/Monaco/Schmiedel datasets, we found that *TWF1* expression in NK cells was the highest, followed by basophils (**Figure S3B**).

**Figure 1:**
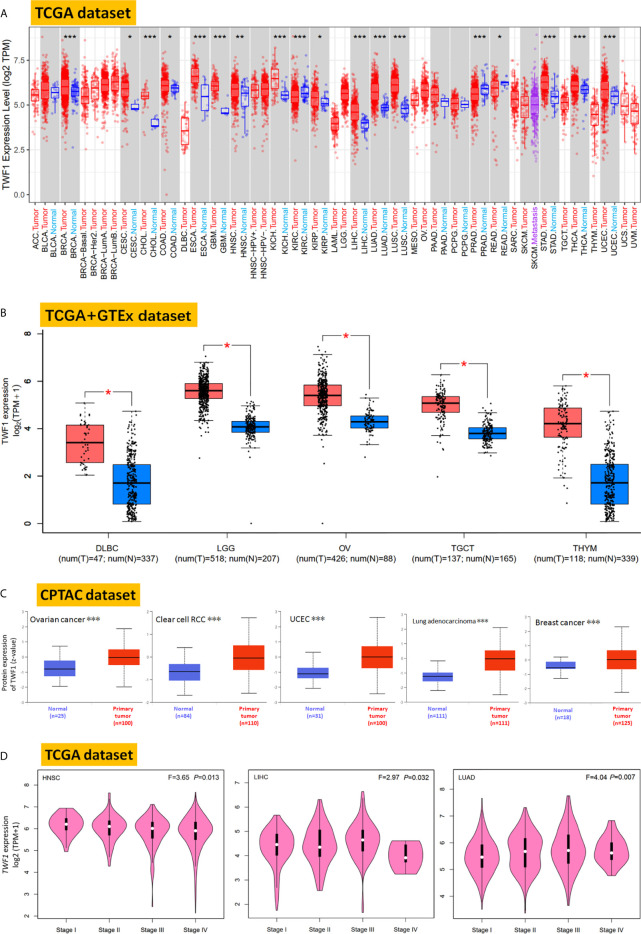
Expression and protein level of *TWF1* in human tumors. **(A)** Expression level of *TWF1* in TCGA tumors *vs.* adjacent tissues (if available) as visualized by TIMER2. *P < 0.05; **P < 0.01; ***P < 0.001. **(B)** Box plot representation of *TWF1* expression level comparison in DLBC, LGG, OV, TGCT and THYM (TCGA project) relative to the corresponding normal tissues (GTEx database). *P < 0.05. **(C)** Total protein level of TWF1 in normal tissue and primary ovarian cancer, clear cell RCC, UCEC, LUAD and breast cancer. Protein data was extracted and analyzed using CPTAC. ***P < 0.001. **(D)** Stage-dependent expression level of *TWF1.* Main pathological stages (stage I, stage II, stage III, and stage IV) of HNSC, LIHC and LUAD were assessed and compared using TCGA data. Expression levels are shown as Log2 (TPM+1).

Next, we used the TIMER2 to study the differential expression of *TWF1* between tumor and adjacent normal tissues for tumors represented in the TCGA repository. As shown in **Figure 1A**, the expression level of *TWF1* in the tumor tissues of BRCA, CHOL (Cholangiocarcinoma), ESCA (Esophageal carcinoma), GBM (Glioblastoma Multiforme), KICH (Kidney Chromophobe), LIHC, LUAD, LUSC (Lung squamous cell carcinoma), STAD (Stomach adenocarcinoma), THCA, UCEC (Uterine Corpus Endometrial carcinoma) (*P*<0.001), HNSC (Head and Neck squamous cell carcinoma) (*P*<0.01), CESC (Cervical squamous cell carcinoma and Endocervical adenocarcinoma), COAD (Colon adenocarcinoma), KIRP (Kidney Renal Papillary Cell carcinoma) (*P*<0.05) is higher than the corresponding control tissues. Remarkably, only few tumor types showed no differential expression (e.g. BLCA (Bladder Urothelial carcinoma), PAAD (Pancreatic adenocarcinoma), PCPG (Pheochromocytoma and Paraganglioma). In contrast, *TWF1* showed lower expression in KIRC (Kidney Renal Clear Cell carcinoma), PRAD (Prostate Adenocarcinoma) (*P*<0.001), and READ (Rectum adenocarcinoma) (*P*<0.05) relative to the corresponding control tissues.

In case of tumors, where normal tissue data was not available in TCGA, we further assessed the expression differences of *TWF1* between the tumor and normal tissues using the GTEx dataset. We found that DLBC, LGG, OV, TGCT and THYM (Thymoma) showed a higher expression in tumor tissues (**Figure 1B**, *P*<0.05). For other tumors, such as ACC (Adrenocortical carcinoma), LAML (Acute Myeloid Leukemia), SARC (Sarcoma), SKCM (Skin Cutaneous Melanoma) or UCS (Uterine Carcinosarcoma), we did not get a significant difference (**Figure S4**). Overall, we found that in the majority of human tumors the *TWF1* expression was elevated.

In addition to transcription, we also assessed TWF1 at a protein level using the large-scale proteome data available through the National Cancer Institute`s CPTAC dataset. We found that the total protein expression of TWF1 was significantly higher in OV, KIRC, UCEC, LUAD, and breast cancer tumor tissues compared to normal tissues (**Figure 1C**, *P*<0.001).

We also used the GEPIA2 tool to analyze the relationship between *TWF1* expression and tumor pathological staging, which indicated stage-specific expressional changes in *TWF1* expression in case of a few tumor types, such as HNSC, LIHC and LUAD (**Figure 1D**, all *P*<0.05), while in most cases we found no clear association (**Figure S5**).

**Figure 2:**
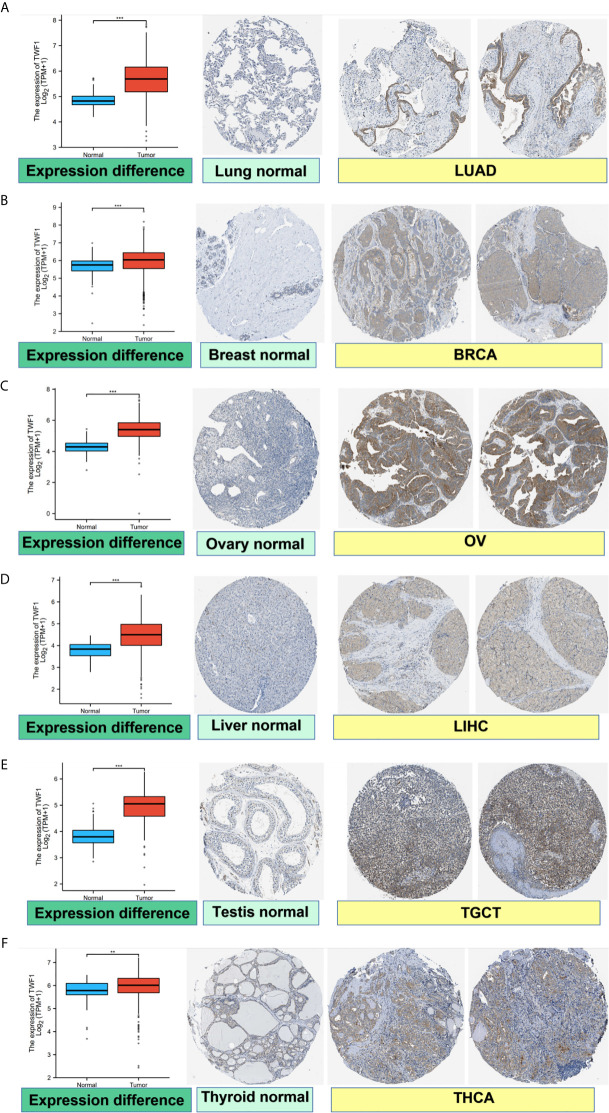
Comparison of *TWF1* gene expression between normal and tumor tissues (left) and immunohistochemistry images in normal (middle) and tumor (right) tissues. TWF1 protein expression was significantly higher in LUAD, BRCA, OV, LIHC, TGCT, THCA. **(A)** Lung. **(B)** Breast. **(C)** Ovary. **(D)** Liver. **(E)** Testis. **(F)** Thyroid. **P < 0.01; ***P < 0.001.

We also analyzed IHC results provided by the HPA database and compared the results with TWF1 gene expression data from TCGA. The results of analysis of data from these two databases were consistent with one another. Normal lung, breast, ovary, liver, testis, and thyroid tissues had negative or medium TWF1 IHC staining, while tumor tissues had medium or strong staining. (**Figures 2A–F**).

### Survival Analysis Data

**Figure 3:**
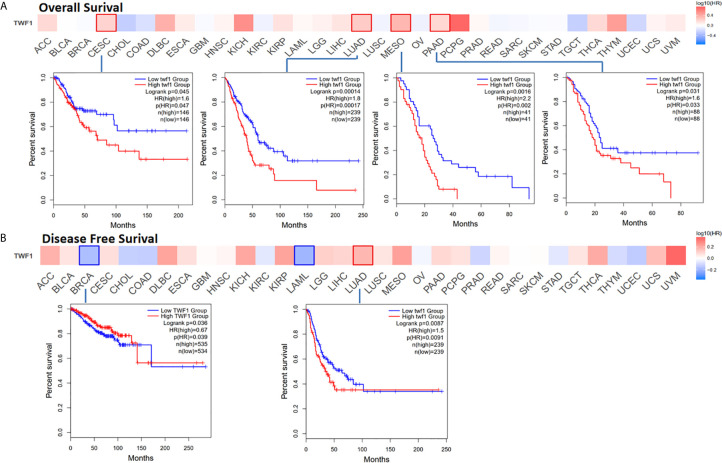
Relationship between *TWF1* expression level and patient survival in TCGA tumors. Relationship between *TWF1* gene expression and survival (overall **(A)**, disease-free survival **(B)** were assessed in all TCGA tumors using GEPIA2. The positive results of survival map and Kaplan-Meier curves are listed.

Next, we wanted to focus on understanding how *TWF1* expression correlate with prognosis and overall survival. We divided cancer cases into high expression group and low expression group according to the expression level of *TWF1*, and then TCGA and GEO datasets were used to study the correlation between *TWF1* expression and prognosis of different tumor patients. High expression of *TWF1* was associated with poor prognosis of OS (Overall survival) for cancers including CESC (*P*=0.045), LUAD (*P*=0.00014), MESO (Mesothelioma) (*P*=0.0016) and PAAD (Pancreatic adenocarcinoma) (*P*=0.031) (**Figure 3A**). DFS (Disease-free survival) analysis data (**Figure 3B**) showed that high expression of *TWF1* is associated with poor prognosis for LUAD (*P*=0.0087). We found that low expression of the *TWF1* was also associated with poor DFS prognosis for BRCA (*P*=0.036).

Using the Kaplan-Meier plotter tool to analyze the survival data, we noted a correlation between high *TWF1* expression level and poor OS, FP (First progression) and PPS (Post-progression survival) prognosis for gastric cancer and lung cancer, poor OS for ovarian cancer, poor RFS (Relapse-free survival) and PPS for breast cancer, and poor PFS (Progression-free survival) for liver cancer (**Figure S6**). Following a meta-analysis, a possible correlation between *TWF1* expression and prognosis of gastric cancer and lung cancer was also detected (*P*<0.00001), while in case of ovarian cancer, breast cancer and liver cancer no association was found (**Figure S7**).

### Genetic Alteration Analysis Data

**Figure 4:**
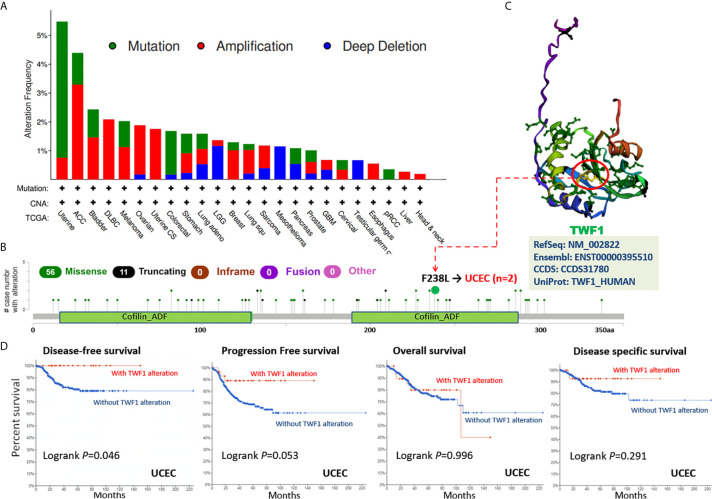
Mutation status of *TWF1* in TCGA tumors. Mutation status of *TWF1* in TCGA tumors was analyzed using the cBioPortal tool. The alteration frequency with mutation type **(A)** and mutation site **(B)** are displayed. **(C)** Mutation site (F238L) within the Cofilin_ADF domain is shown in the 3D structure of TWF1. **(D)** Analysis of the correlation between mutation status and OS (Overall survival), DSS (Disease-specific survival), DFS (Disease-free survival) and PFS (Progression-free survival) of UCEC using the cBioPortal tool.

Human cancers develop due to the accumulation of genetic alterations. Thus, we next wanted to explore the *TWF1* genetic alterations in human tumor samples. According to our analysis, the frequency of *TWF1* alteration (>5%) is the highest in uterine tumors with “mutation” as the primary type. ACC had the highest incidence of “amplification” type of CNA, with the frequency of ~4% (**Figure 4A**). On **Figure 4B**, we show additional mutations and their location within *TWF1*. We found no main type of genetic alteration and their location seemed somewhat sporadic, some falling within the Cofilin-ADF domain. For instance, a missense mutation, F238L alteration, in the Cofilin-ADF domain, was only detected in 2 cases of UCEC. We acquired the F238L site visualized on the 3D structure of TWF1 protein (**Figure 4C**). To see whether there is a relationship between certain genetic alterations of *TWF1* and the clinical survival prognosis of patients, we systematically studied and correlated these in various types of tumors. In UCEC patients with genetic alteration of *TWF1* showed a better prognosis in DFS (*P*=0.046), but not PFS (*P*=0.053), OS (*P*=0.996), and disease specific survival (*P*=0.291), compared with patients without *TWF1* alterations (**Figure 4D**).

We further explored the relationship between *TWF1* expression and TMB (Tumor mutational burden) and MSI (Microsatellite instability) across all tumors represented in TCGA. We noticed that the expression of *TWF1* in ACC is positively correlated with TMB (*P*=0.012), PCPG (*P*=0.013), UCEC (*P*=0.0032), STAD (*P*=5.3e-07) and SKCM (*P*=0.037) (**Figure S8**). The expression of *TWF1* is also positively correlated with MSI in COAD (*P*<0.001), STAD (*P*=4.2e-07), READ (*P*=0.0013) and UCEC (*P*=7.3e-05), but negatively correlated with DLBC (*P*=7.2e-05), LUAD (*P*=0.0017) and PRAD (*P*=6.2e-09) (**Figure S9**). Taken together, these findings suggest that *TWF1* genetic alterations has to be considered as possible drivers of the above listed tumors.

### Protein Phosphorylation Analysis Data

**Figure 5:**
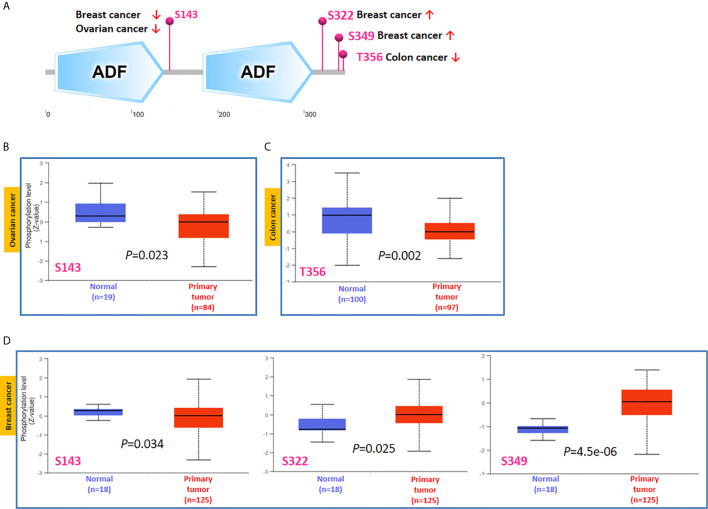
Tumor-associated protein phosphorylation of TWF1. Comparison of the level of TWF1 phosphoproteins (S143, S322, S349 and T356 sites) between normal tissue and primary tissue of selected tumors. **(A)** Phosphoprotein sites detected based on the CPTAC dataset in TWF1 are depicted in a schematic diagram. **(B–D)** Box plot representation of TWF1 phosphoprotein levels in ovarian cancer, colon cancer and breast cancer.

Phosphorylation-dephosphorylation cascade is known to be a key event in oncogenesis. TWF1 is undergoing several posttranslational modifications, including phosphorylation of serine and threonine residues. Thus, we next compared the phosphorylation of TWF1 between normal and primary tumor tissues. We analyzed three kinds of tumors (breast cancer, ovarian cancer and colon cancer) in more details using the CPTAC dataset. As summarized in **Figure 5A**, we found that the phosphorylation level of S143 of TWF1 in primary tumor tissues of ovarian cancer and breast cancer is significantly reduced (**Figures 5B, D**). We found lower phosphorylation level of the T356 for colon cancer, and increased phosphorylation level of the S322 and S349 for breast cancer (**Figures 5C, D**).

### Immune Infiltration Analysis Data

**Figure 6:**
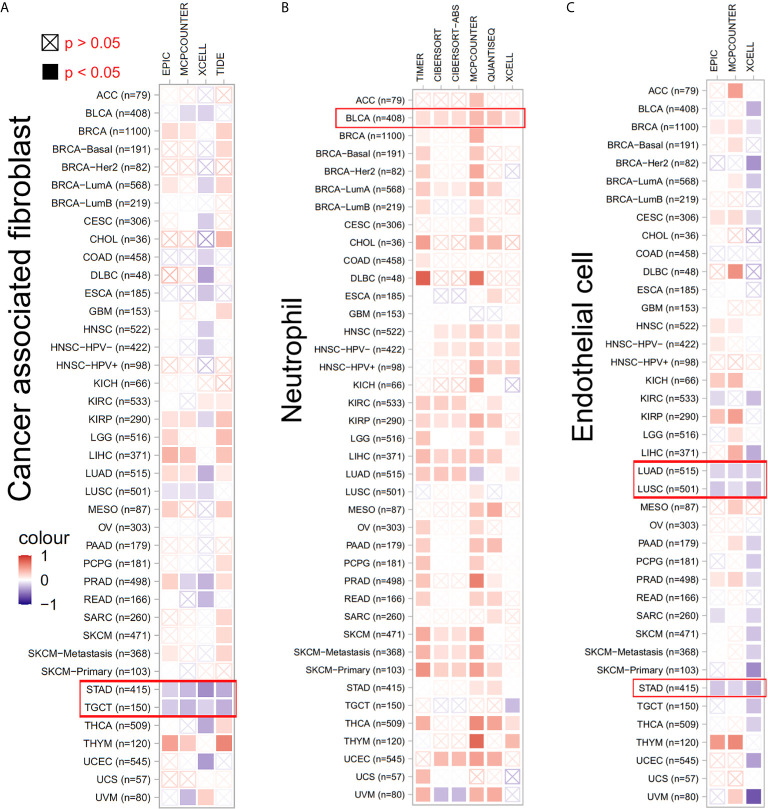
The correlation between *TWF1* expression level and infiltration of cancer-associated fibroblasts, neutrophils and endothelial cells. **(A–C)** TIMER, CIBERSORT, CIBERSORT-ABS, TIDE, XCELL, MCPCOUNTER, QUANTISEQ and EPIC algorithms were used for the correlative analysis of the level of cancer-associated infiltration of fibroblasts, neutrophils and endothelial cells and the expression levels of the *TWF1* gene across all tumors in TCGA. The red color indicates a positive correlation (0–1), while the blue color represents a negative correlation (−1–0). The correlation with *P*-value <0.05 is considered as statistically significant. Statistically non-significant correlations values are marked with a cross.

Considering the role of TWF1 in the regulation of actin cytoskeleton structure, and the known role of actin cytoskeleton in cell migratory processes, we hypothesized that altered *TWF1* expression level or its genetic alteration may impact the tumor infiltrating immune cell response (18–20). Thus, we applied the TIMER, CIBERSORT, CIBERSORT-ABS, TIDE, XCELL, MCPCOUNTER, QUANTISEQ and EPIC algorithms to explore the correlation between the infiltration level of different immune and endothelial cells and *TWF1* expression in multiple tumor types of TCGA. Interestingly, we discovered a negative correlation of *TWF1* expression and the estimated infiltration value of cancer-associated fibroblasts for the STAD and TGCT. *TWF1* expression and the endothelial cell infiltration for the LUAD, LUSC and STAD also showed negative correlation, while a positive correlation was found between the neutrophil cells and *TWF1* expression in the tumors of BLCA (**Figure 6**).

### Enrichment Analysis of TWF1-Related Partners

**Figure 7:**
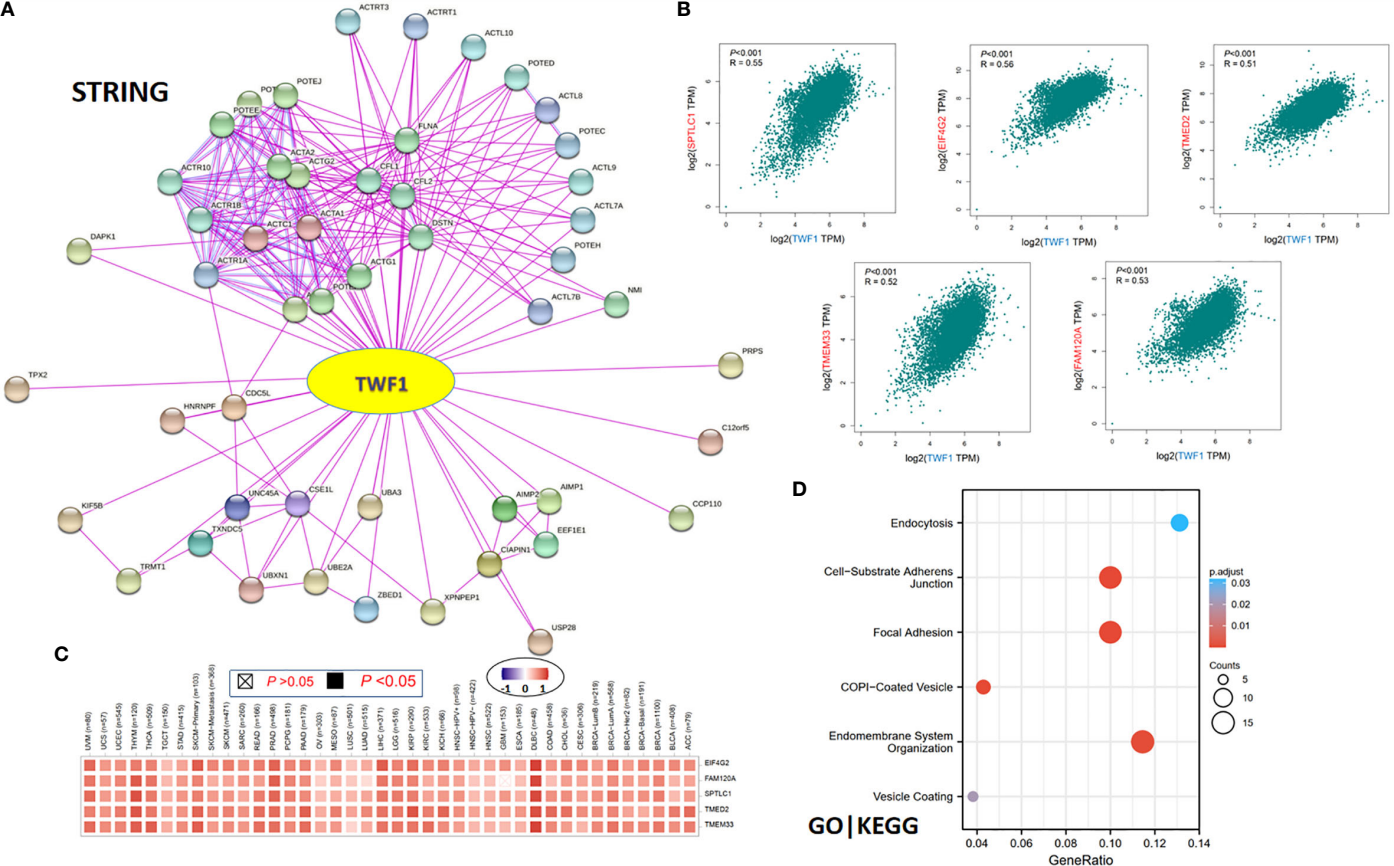
TWF1-related gene enrichment and pathway analysis. **(A)** STRING protein network map of experimentally determined TWF1-binding proteins. Colored nodes indicate the individual proteins identified. **(B)** Expression correlation between *TWF1* and representative genes (*SPTLC1*, *EIF4G2*, *TMED2*, *TMEM33* and *FAM120A*) of the top *TWF1*-correlated genes in TCGA projects as determined by GEPIA2. **(C)** Heatmap representation of the expression correlation data between *TWF1* and *SPTLC1*, *EIF4G2*, *TMED2*, *TMEM33* and *FAM120A* in the TCGA tumors. **(D)** GO|KEGG pathway analysis based on the TWF1-binding and interacted genes.

Finally, in order to study the molecular mechanism of the *TWF1* gene in tumorigenesis and development, we filtered out the known TWF1-interacting proteins and the *TWF1* expression-correlated genes for a series of pathway enrichment analyses. Using the STRING tool, we acquired a total of 50, experimentally detected TWF1-binding proteins. **Figure 7A** shows the interaction network of these 50 proteins. Then we used the GEPIA2 tool to combine all tumor expression data of TCGA and acquired the top 100 genes which correlated with *TWF1* expression. The expression of *TWF1* was positively associated with that of *SPTLC1* (Serine palmitoyl transferase long chain base subunit 1) (*R*=0.55), *EIF4G2* (Eukaryotic translation initiation factor 4 gamma 2) (*R*=0.56), *TMED2* (Transmembrane p24 trafficking protein 2) (*R*=0.51), *TMEM33* (Transmembrane Protein 33) (*R*=0.52) and *FAM120A* (Family With Sequence Similarity 120A) (*R*=0.53) genes (all *P*<0.001) (**Figure 7B**). Heatmap data displayed that *TWF1* had a strong positive correlation with the five genes above in most cancer types (**Figure 7C**).

We combined the two datasets to perform GO and KEGG enrichment analyses. The GO|KEGG pathway analysis revealed “focal adhesion” and “vesicle transportation” among the top hits, suggesting these pathways in the effect of TWF1 on tumor pathogenesis (**Figure 7D**).

## Discussion

Previous studies have shown that the multifunctional TWF1 protein is involved in a series of fundamental, cross-species cell biology processes in healthy and diseased conditions. TWF1 regulates the cytoskeleton through direct interaction with actin filaments (11). Its overexpression also enhances the EMT (Epithelial-mesenchymal transition) of breast cancer cells by increasing cytoskeleton dynamics and activating mesenchymal lineage transcription factors, such as MKL1 and SRF (21). It has also been described to enhance the expression of cyclin D1 in bovine mammary epithelial cells and luminal A/B breast cancer cells (22, 23). In our study, “HomoloGene” and phylogenetic tree analysis data confirmed the conservation of TWF1 protein structure across different species, however additional gain- and loss-of-function studies will be needed to further explore its function in different cellular context.

Growing number of studies have focused on the analysis of the function of TWF1 in diseases, including cancer. Nevertheless, it remains poorly understood whether TWF1 is involved in the oncogenesis of certain tumor types, or rather play a role in more common pathways driving tumor pathogenesis. Thus, here we set out to perform a pan-cancer analysis of TWF1. Our comprehensive approach included the exploration of the *TWF1* gene expression in a total of 33 different tumors based on the data of TCGA. We also systematically collected and integrated protein and phosphor-protein data using CPTAC and GEO databases, as well as other molecular features and genetic alterations.

In our result, the expression level of *TWF1* in the tumor tissues of BRCA, CHOL, ESCA, GBM, KICH, LIHC, LUAD, LUSC, STAD, THCA, UCEC, HNSC, CESC, COAD, and KIRP is higher than the corresponding control tissues, whereas low expression was observed in KIRC, PRAD, and READ. The difference in *TWF1* expression levels in different tumor types may reflect distinct underlying functions and mechanisms. We further found that over-expression of *TWF1* generally predicted poor OS for patients with tumors with high *TWF1* expression, such as CESA, LUAD, MESO, and PAAD. These results suggest that *TWF1* is a potential biomarker for predicting the prognosis of tumor patients.

We have made several notable findings in our analysis. Especially in MESO, we found that high expression of *TWF1* was associated with poor prognosis of OS for MESO (*P*=0.0016). The role of TWF1 in mesothelioma was rarely reported. Our findings may provide a novel clinical biomarker for predicting overall survival of mesothelioma patients.

Regarding lung cancer, we explored the datasets of the TCGA-LUSC (n=501) and TCGA-LUAD (n=515) and found a correlation between the high expression of *TWF1* and late clinical staging (*P*<0.01) poor OS prognosis (*P*<0.001), poor DFS (*P*<0.01) specific for LUAD but not for LUSC. Indeed, significant association between high *TWF1* expression in LUAD tissues with poor TNM stage (*P*=0.0106), more lymph node metastasis (*P*<0.001), larger tumor size (*P*=0.035) and late clinical staging (*P*=0.014) has been previously described (24).

Based on our analysis, the expression level of *TWF1* and total protein of TWF1 in the tumor tissues of BRCA is higher than the corresponding control tissues. In addition, the GO|KEGG pathway analysis revealed “focal adhesion” among the top hits. Recent studies have reported that TWF1 has a close association with breast cancer, and miR-30c has been proposed to directly target *TWF1*, leading to inhibition of chemotherapy resistance and inhibit tumor invasion of human breast tumor (13, 25). *TWF1* depletion in breast cancer cell line MDA-MB-231 cells drastically reduced vinculin positive focal adhesions, suppressed organization of F-actin, and enhanced the EMT wherein cells acquire spherical morphology (13). Those results show that TWF1 can play an important role in BRCA.

Our TCGA-based survival analysis results also indicated a correlation between *TWF1* high expression and poor OS for PAAD. Hua et al. reported that *TWF1* is expressed higher in gemcitabine resistant pancreatic cancer cells relative to parent cells *in vitro* (26). Sun et al. also found that miR-30c inhibits pancreatic cancer cell proliferation by targeting *TWF1* and indicates a poor prognosis (27).

In this study, we presented evidence for the correlation between *TWF1* expression and MSI or TMB. Moreover, our findings suggest a statistical positive correlation between *TWF1* expression and the neutrophil in the tumors of BLCA, negative correlation for endothelial cell in the LUAD, LUSC and STAD, negative correlation for cancer-associated fibroblasts in STAD and TGCT using multiple immune deconvolution methods to evaluate.

In summary, from our comprehensive pan-cancer analysis of *TWF1*, we found a statistical association between *TWF1* expression and clinical prognosis, protein phosphorylation, immune cell infiltration, tumor mutation burden, or microsatellite instability for a variety of human cancers, contributing to clarify the role of TWF1 in tumorigenesis from a variety of perspectives.

## Data Availability Statement

The datasets presented in this study can be found in online repositories. The names of the repository/repositories and accession number(s) can be found in the article/**Supplementary Material**.

## Author Contributions

PC contributed to the concept. GH, YW, and JLC wrote the manuscript. YS, CZ, and RZ participated in the study design and helped draft the manuscript. GH, FZ, JFC, WDC, and WMC performed the literature search and collected the data. GH and HG analyzed the data. GH, YW, and JLC contributed equally to this study. All authors contributed to the article and approved the submitted version.

## Funding

This work was supported by funding from the Tianjin major disease prevention and control science and Technology project, Tianjin municipal science and technology bureau (18ZXDBSY00050).

## Conflict of Interest

The authors declare that the research was conducted in the absence of any commercial or financial relationships that could be construed as a potential conflict of interest.

## Supplementary Material

Supplementary Figure 1 Structural characteristics of TWF1 in different species. **(A)** Human *TWF1* gene location in hg38; **(B)** Conserved protein domains of TWF1 across different species.

Supplementary Figure 2 Phylogenetic tree of TWF1. Phylogenetic tree of TWF1 was constructed based on NCBI taxonomy browser, displaying 20 species.

Supplementary Figure 3 Expression level of *TWF1* in different cells and tissues under normal physiological conditions. **(A)** Expression of *TWF1* gene in different tissues based on HPA, GTEx and FANTOM5 database; **(B)** Expression of *TWF1* gene in different blood cell types based on the HPA, Monaco and Schmiedel datasets.

Supplementary Figure 4 Comparison of *TWF1* expression levels using GTEx database. The expression statuses of the *TWF1* gene in tumors without available appropriate control in TCGA, such as ACC, LAML, SARC, SKCM, UCS, were compared to normal tissues based on the GTEx databases.

Supplementary Figure 5 Relationship between *TWF1* expression level and pathological stages of TCGA tumors. Pathological stage plot derived for *TWF1* gene expression data in GEPIA2 for ACC, BLCA, BRCA, CHOL, CESC, COAD, DLBC, ESCA, KICH, KIRC, KIRP, LUSC, OV, PAAD, READ, SKCM, STAD, TGCT, THCA, UCEC and UCS.

Supplementary Figure 6 Using Kaplan-Meier plotter to study the relationship between *TWF1* gene expression and tumor prognosis. Kaplan-Meier plotter was used to perform the survival analyses (OS, DMFS, RFS, PFS, PPS, FP, and DSS) in gastric cancer **(A)**, lung cancer **(B)**, ovarian cancer **(C)**, breast cancer **(D)**, and liver cancer **(E)** patients.

Supplementary Figure 7 Correlation between *TWF1* expression and cancer prognosis using meta-analysis. Meta-analysis was conducted for pooling of a series of survival analysis by the expression level of the* TWF1 *gene in ovarian cancer, lung cancer, gastric cancer, breast cancer, and liver cancer patients.

Supplementary Figure 8 Correlation between *TWF1* expression and tumor mutational burden. Correlation analysis was performed between *TWF1* expression and tumor mutational burden (TMB) based on the TCGA project data. The *P*-value and the partial correlation values of +0.28 and −0.28 are displayed.

Supplementary Figure 9 Correlation between *TWF1* expression and microsatellite instability. Correlation analysis was performed between *TWF1* expression and microsatellite instability (MSI) using data from the TCGA project. The *P*-value and the partial correlation values of +0.54 and −0.54 are displayed.
