# Supplementary material for: A Pan-Cancer Analysis of the Oncogenic Role of Twinfilin Actin Binding Protein 1 in Human Tumors
Source: Front Oncol. 2021 May 25;11:692136. doi: 10.3389/fonc.2021.692136 (PMC8185641; doi:10.3389/fonc.2021.692136)
Supplement: Supplementary Figure 1 — Structural characteristics of TWF1 in different species. (A) Human TWF1 gene location in hg38; (B) Conserved protein domains of TWF1 across different species. [file Image_1.pdf]

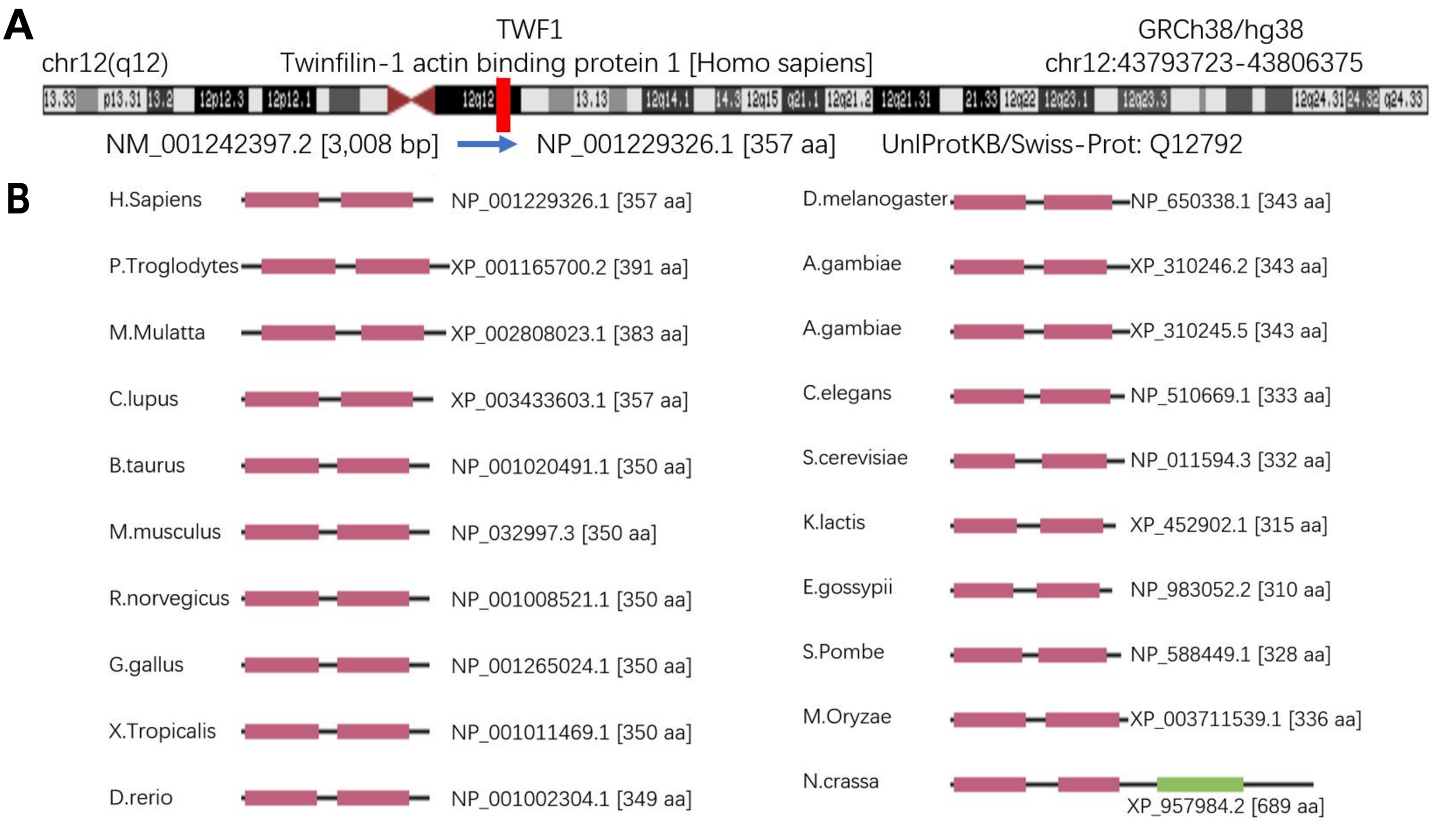

Conserved Domains

- PIPKc (cl02572): Phosphatidylinositol phosphate kinases (PIPK) catalyze.
- ADF_gelsolin (cl15697): Actin depolymerization factor/cofilin- and gelsolin-like domains.

Supplementary Figure 1

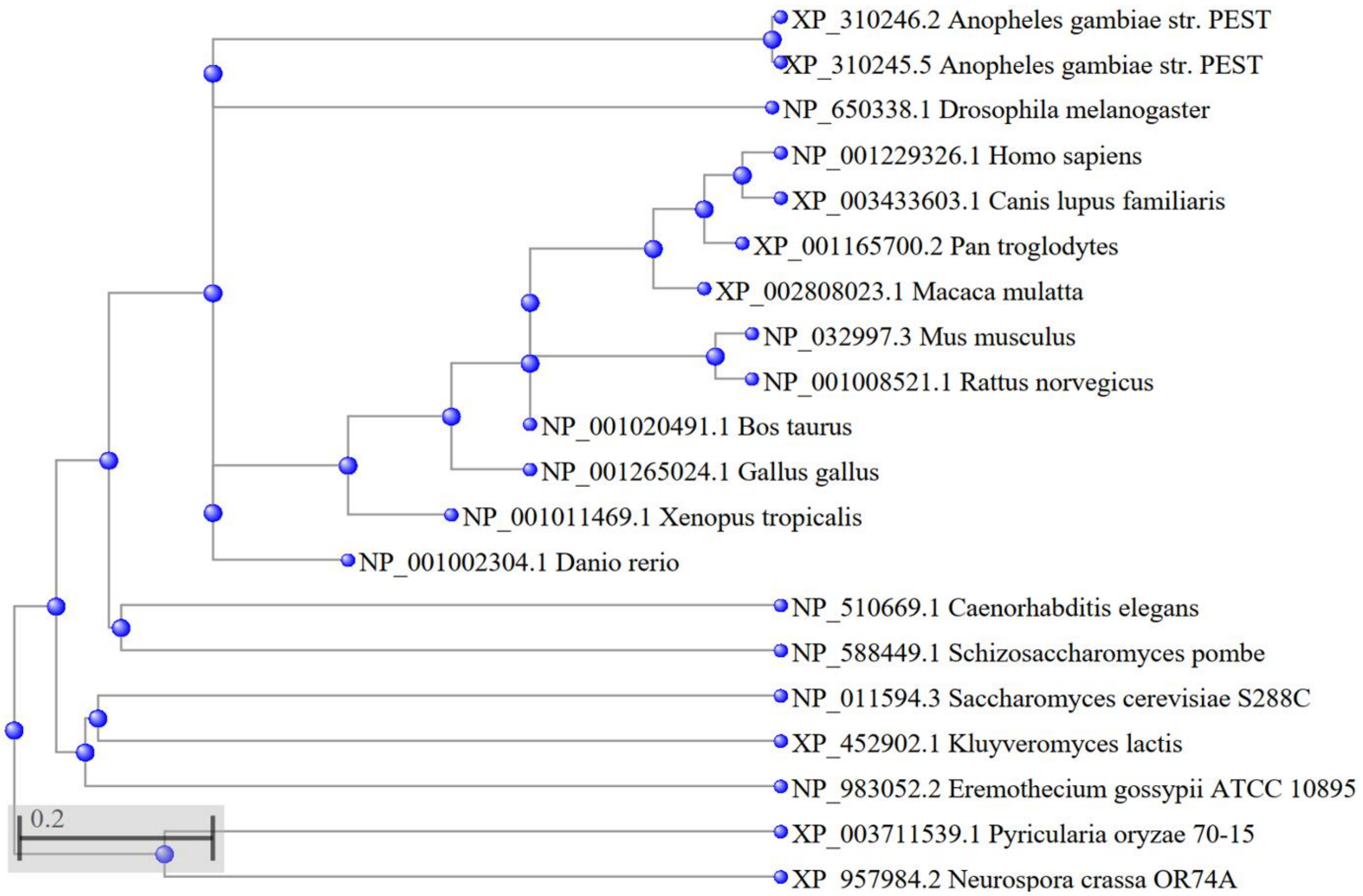

Supplementary Figure 2

A

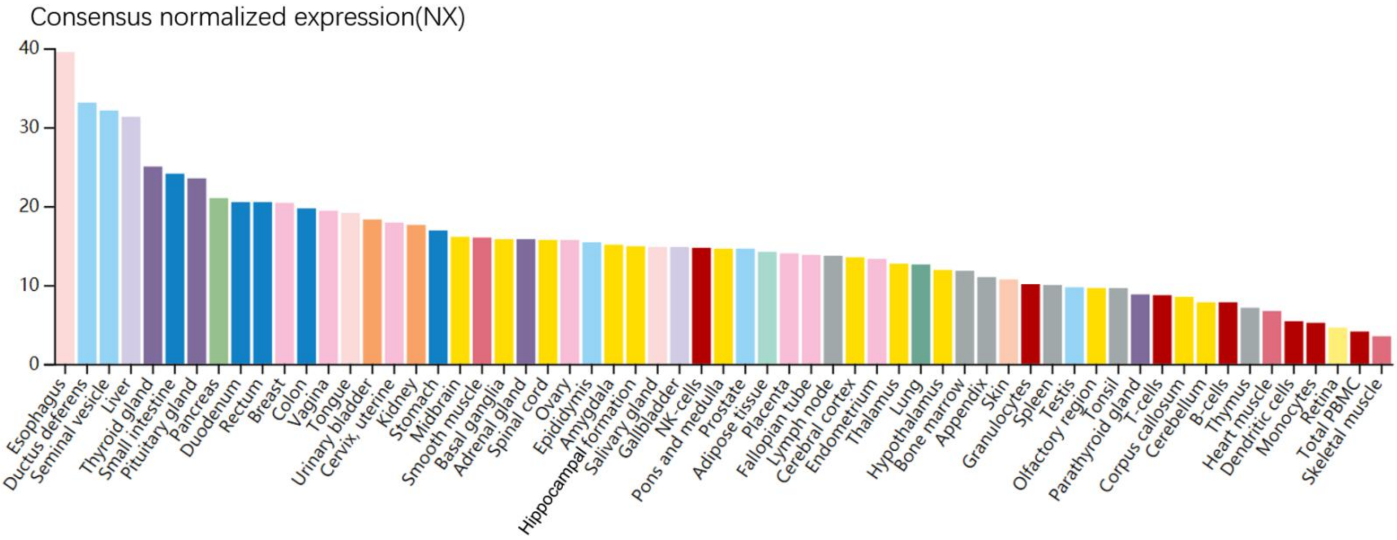

B

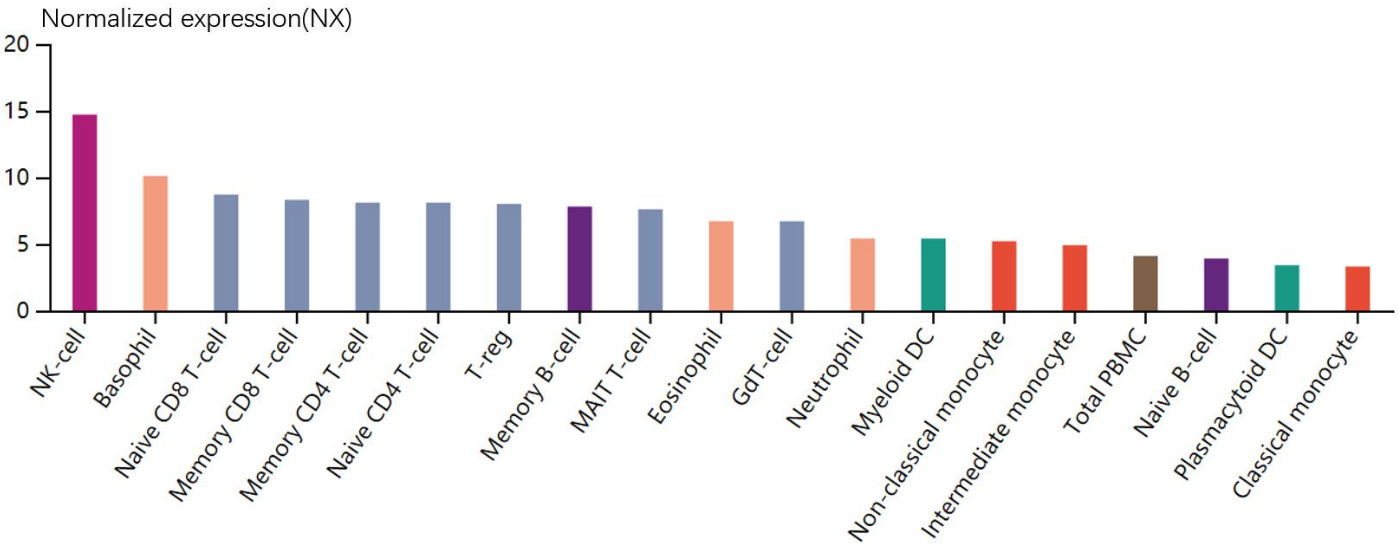

Supplementary Figure 3

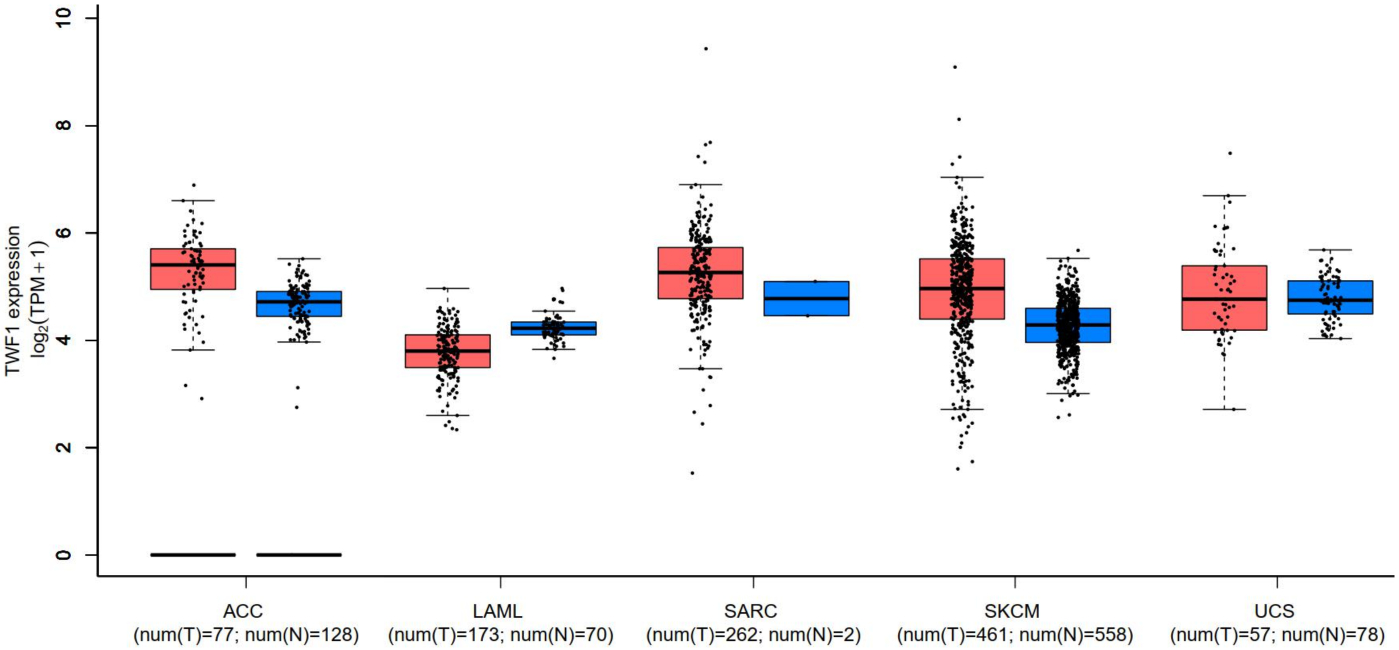

Supplementary Figure 4

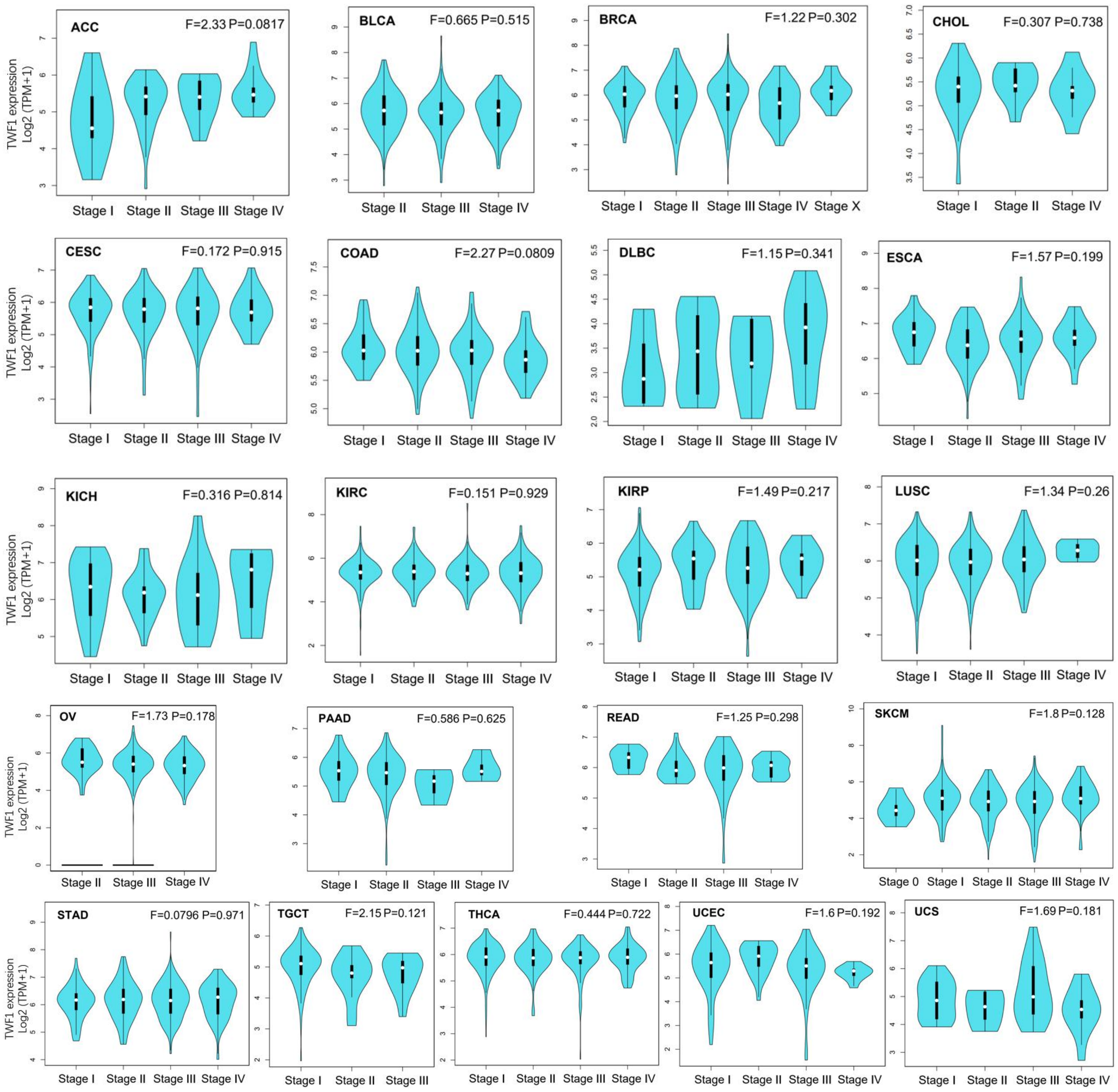

Supplementary Figure 5

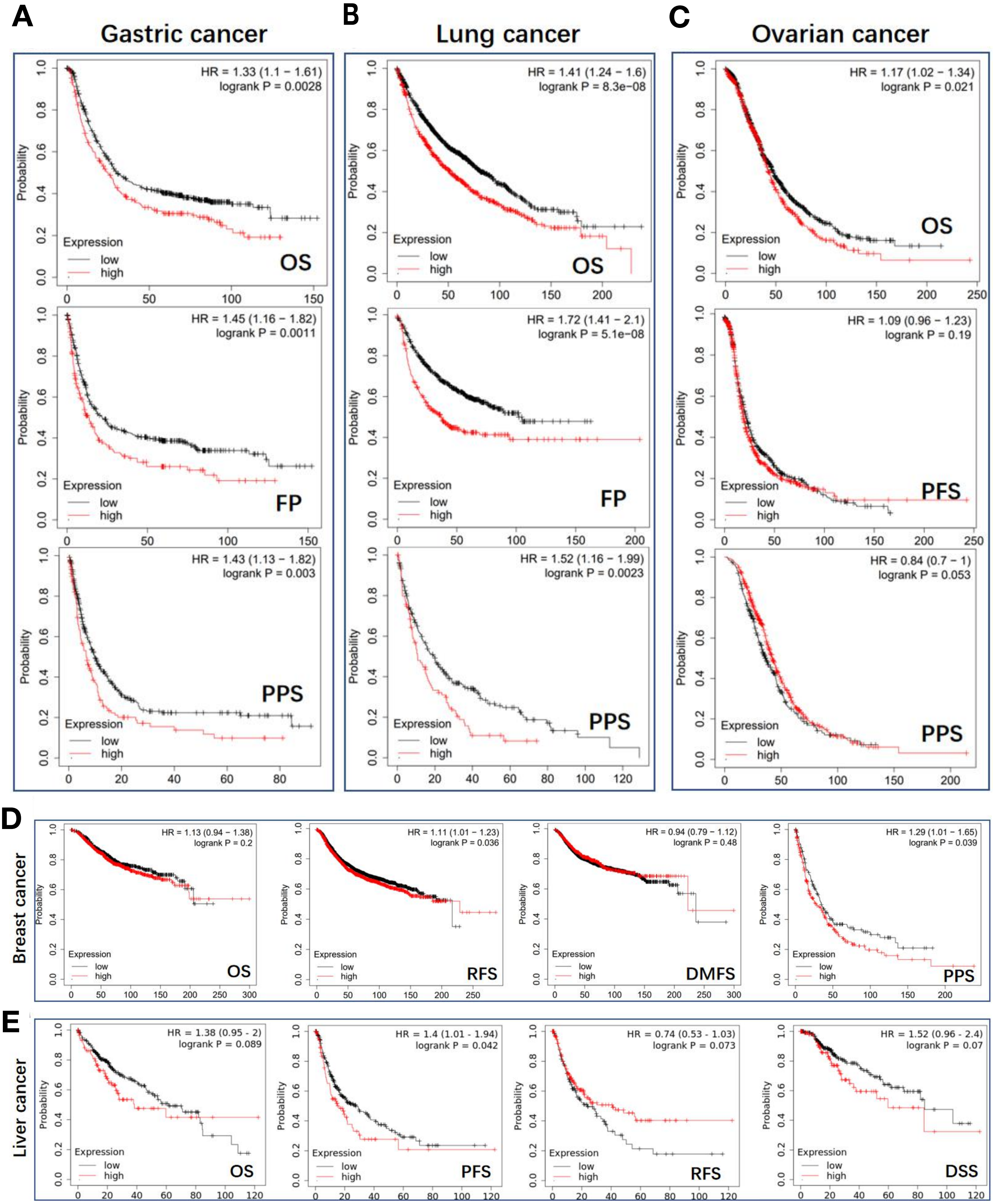

Supplementary Figure 6

Study or Subgroup	log[Hazard Ratio]	SE	Weight	Hazard Ratio IV, Random, 95% CI
**Gastric cancer**				
Gastric cancer-OS	0.12385164	0.04220234	6.4%	1.13 [1.04, 1.23]
Gastric cancer-FP	0.161368	0.04990138	5.8%	1.18 [1.07, 1.30]
Gastric cancer-PPS	0.15533604	0.05280432	5.6%	1.17 [1.05, 1.30]
**Subtotal (95% CI)**			**17.9%**	**1.15 [1.09, 1.22]**
Heterogeneity: Tau² = 0.00; Chi² = 0.40, df = 2 (P = 0.82); I² = 0%				
Test for overall effect: Z = 5.23 (P < 0.00001)				
**Lung cancer**				
Lung cancer-OS	0.14921911	0.02823936	7.5%	1.16 [1.10, 1.23]
Lung cancer-FP	0.23552845	0.0441327	6.3%	1.27 [1.16, 1.38]
Lung cancer-PPS	0.18184359	0.05979466	5.1%	1.20 [1.07, 1.35]
**Subtotal (95% CI)**			**18.9%**	**1.20 [1.13, 1.26]**
Heterogeneity: Tau² = 0.00; Chi² = 2.73, df = 2 (P = 0.26); I² = 27%				
Test for overall effect: Z = 6.53 (P < 0.00001)				
**Ovarian cancer**				
Ovarian cancer-OS	0.06818586	0.03023077	7.3%	1.07 [1.01, 1.14]
Ovarian cancer-PFS	0.0374265	0.02745762	7.5%	1.04 [0.98, 1.10]
Ovarian cancer-PPS	-0.07572071	0.03951581	6.6%	0.93 [0.86, 1.00]
**Subtotal (95% CI)**			**21.5%**	**1.01 [0.94, 1.09]**
Heterogeneity: Tau² = 0.00; Chi² = 8.74, df = 2 (P = 0.01); I² = 77%				
Test for overall effect: Z = 0.37 (P = 0.71)				
**Breast cancer**				
Breast cancer-OS	0.05307844	0.04253858	6.4%	1.05 [0.97, 1.15]
Breast cancer-RFS	0.04532298	0.02183259	7.9%	1.05 [1.00, 1.09]
Breast cancer-DMFS	-0.02687215	0.03867116	6.7%	0.97 [0.90, 1.05]
Breast cancer-PPS	0.11058971	0.05437821	5.5%	1.12 [1.00, 1.24]
**Subtotal (95% CI)**			**26.5%**	**1.04 [0.99, 1.09]**
Heterogeneity: Tau² = 0.00; Chi² = 4.83, df = 3 (P = 0.18); I² = 38%				
Test for overall effect: Z = 1.69 (P = 0.09)				
**Liver cancer**				
Liver cancer-OS	0.13987909	0.08247612	3.7%	1.15 [0.98, 1.35]
Liver cancer-PFS	0.14612804	0.07231642	4.3%	1.16 [1.00, 1.33]
Liver cancer-RFS	-0.13076828	0.07361259	4.2%	0.88 [0.76, 1.01]
Liver cancer-DSS	0.18184359	0.10151531	2.9%	1.20 [0.98, 1.46]
**Subtotal (95% CI)**			**15.2%**	**1.08 [0.93, 1.26]**
Heterogeneity: Tau² = 0.02; Chi² = 10.48, df = 3 (P = 0.01); I² = 71%				
Test for overall effect: Z = 1.04 (P = 0.30)				
**Total (95% CI)**			**100.0%**	**1.09 [1.05, 1.14]**
Heterogeneity: Tau² = 0.01; Chi² = 66.44, df = 16 (P < 0.00001); I² = 76%				
Test for overall effect: Z = 4.09 (P < 0.0001)				
Test for subgroup differences: Chi² = 22.77, df = 4 (P = 0.0001), I² = 82.4%				

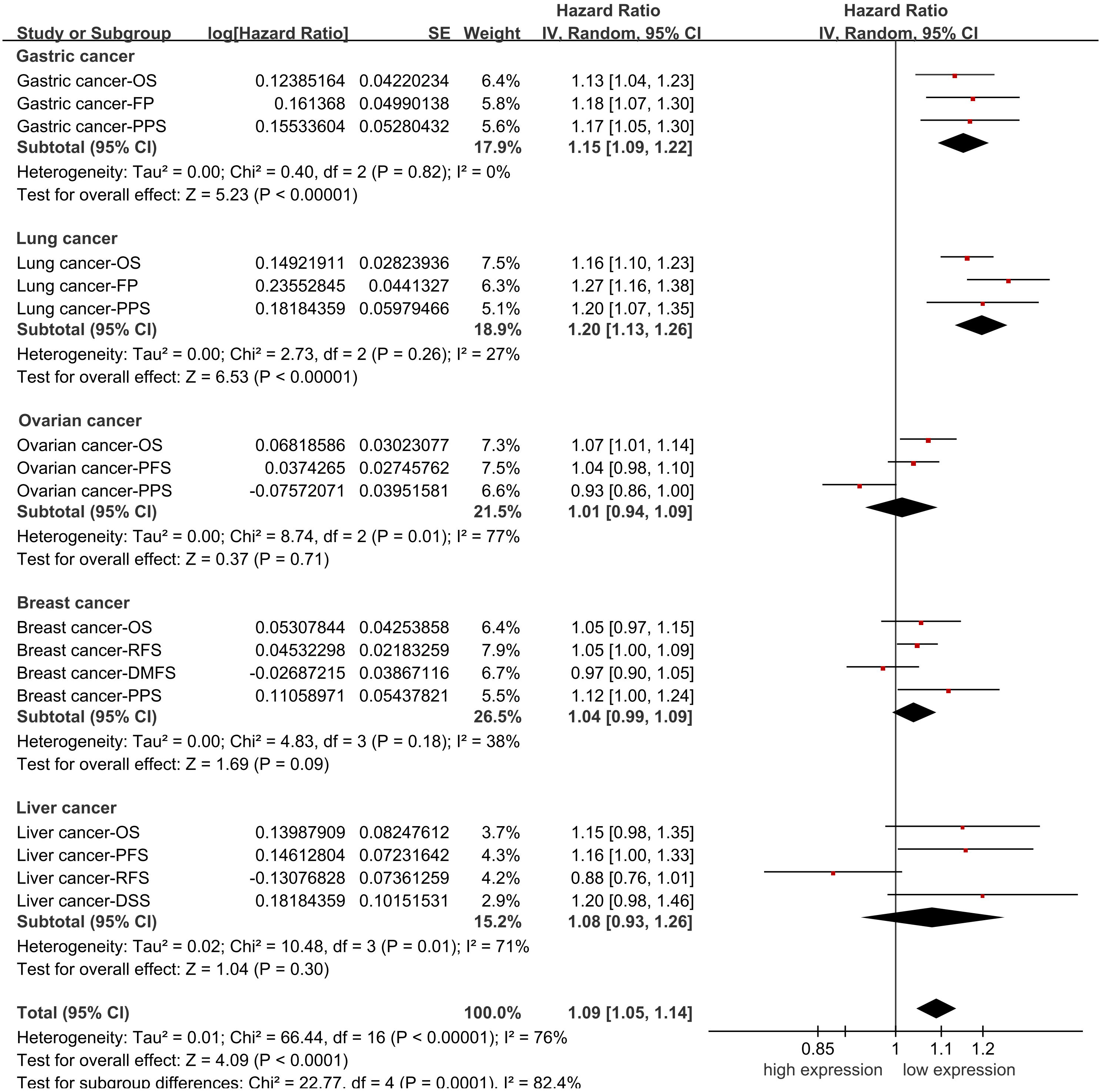

Supplementary Figure 7

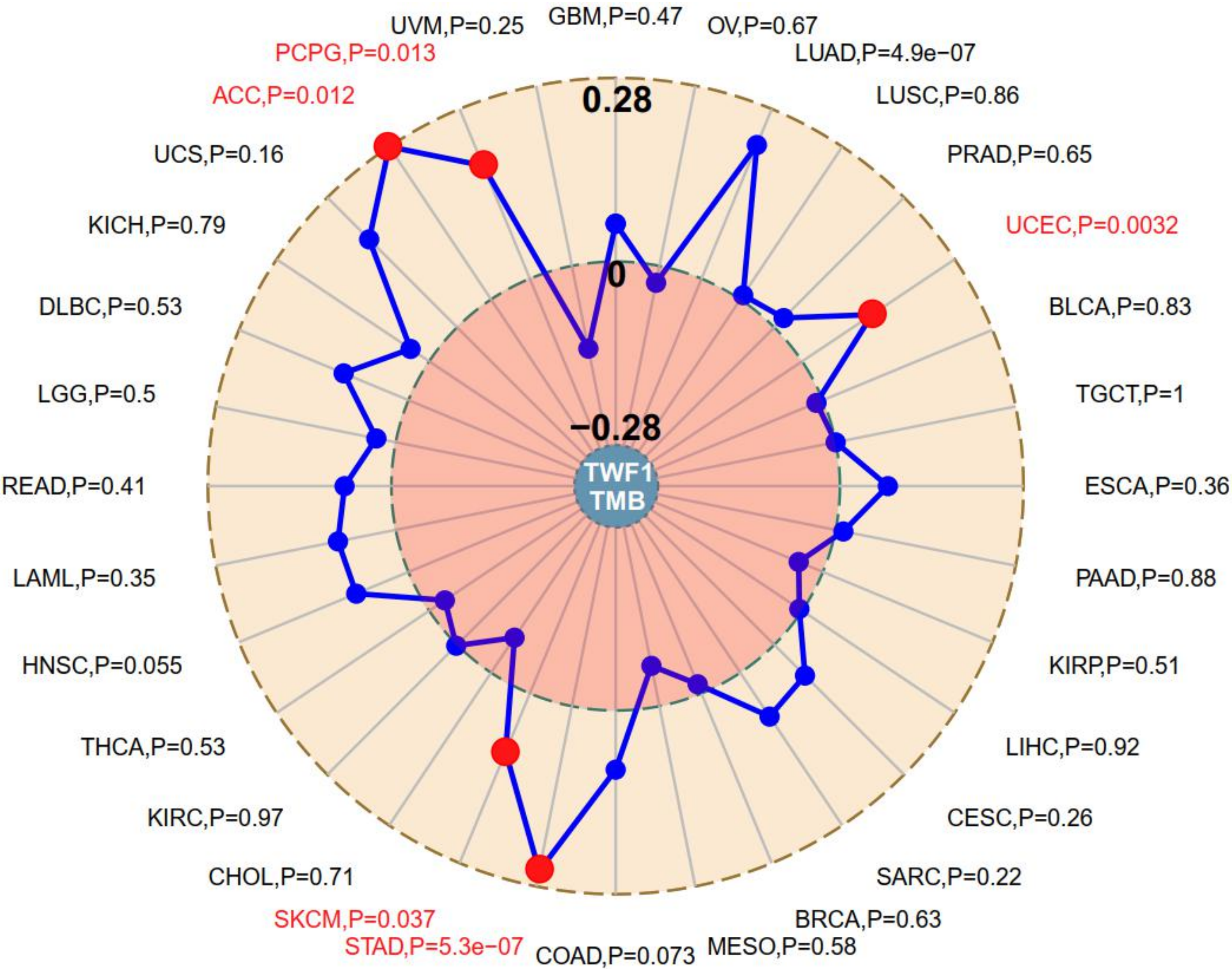

Supplementary Figure 8

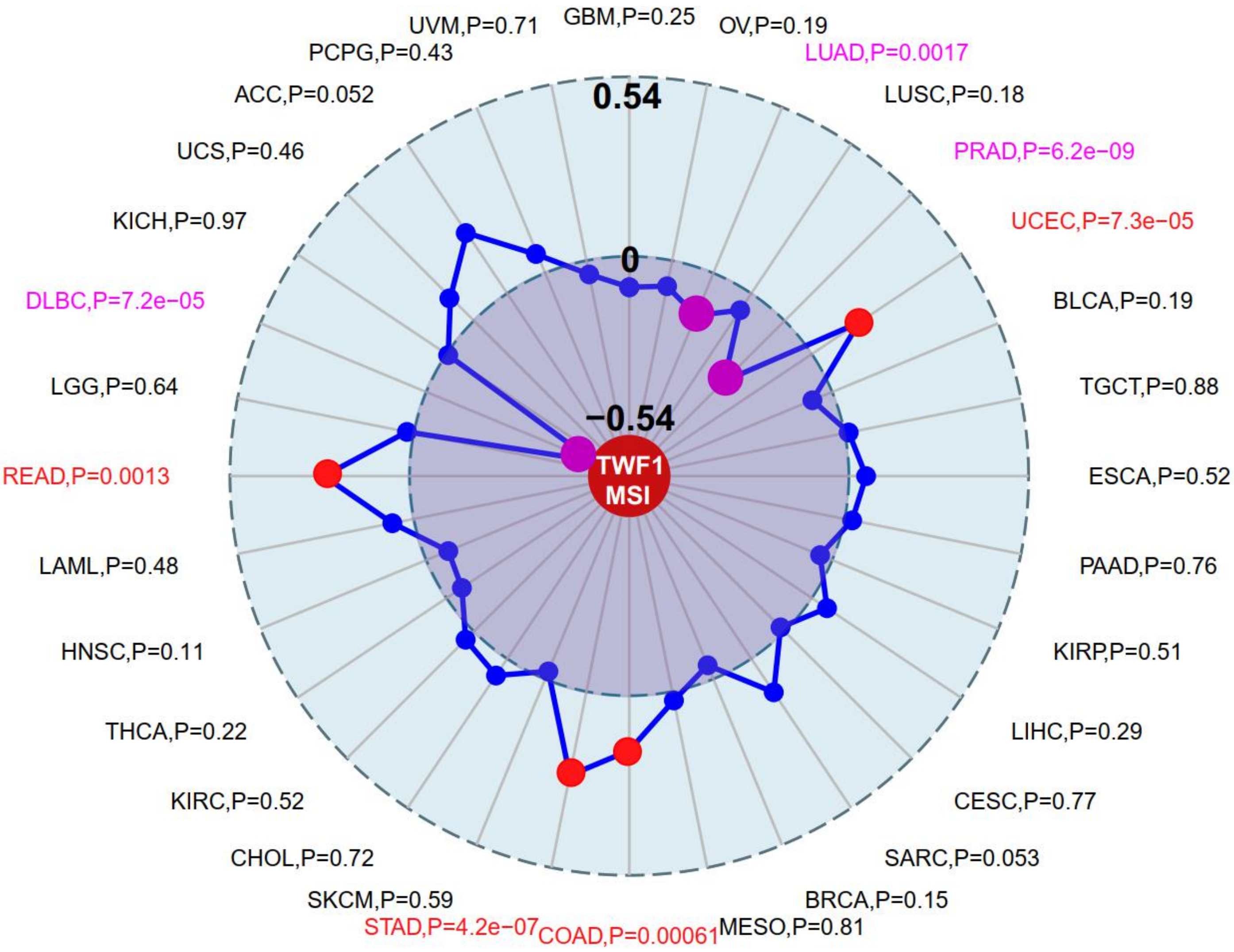

Supplementary Figure 9
