# Supplementary material for: A Pan-Cancer Analysis of the Oncogenic Role of Twinfilin Actin Binding Protein 1 in Human Tumors
Source: Front Oncol. 2021 May 25;11:692136. doi: 10.3389/fonc.2021.692136 (PMC8185641; doi:10.3389/fonc.2021.692136)
Supplement: Supplementary file 2 [file DataSheet_1.docx]

**A pan-cancer analysis of the oncogenic role of Twinfilin Actin Binding Protein 1 in** **human tumors**

**Supplementary data**

**Supplementary materials and methods**

***1. Gene mapping and protein structure analysis***

The *TWF1* genome location information was obtained from UCSC human genome browser (GRCh38/hg38) (<http://genome.ucsc.edu/>) (1). Conserved functional domain analysis and the phylogenetic tree of TWF1 in different species was conduct in NCBI (National Center for Biotechnology Information) (https://www.ncbi.nlm.nih.gov/).

***2. Gene expression analysis of HPA***

The expression level of the *TWF1* under physiological conditions in different cell- and tissue types was analyzed using HPA (Human Protein Atlas) database (<https://www.proteinatlas.org/humanproteome/pathology>). “Low specificity” was defined by “NX (Normalized expression) ≥1 in at least one tissue/region/cell type but not elevated in any tissue/region/cell type”. The detailed information can be found in [https://www.proteinatlas.org/ENSG00000197157-TWF1](https://www.proteinatlas.org/ENSG00000197157-SND1).

1. ***Survival prognosis analysis of Kaplan-Meier plotter***

Kaplan-Meier plotter (<http://kmplot.com/analysis/>) was used to pool the different GEO datasets for a series of analyses of OS, DMFS (distant metastasis-free survival), RFS (relapse-free survival), PPS (post-progression survival), FP (first progression), DSS (disease-specific survival), and PFS (progress-free survival). The “autoselect best cutoff”parameter was used to distinguish between high and low expression groups. The hazard ratio (HR), 95% confidence intervals and log-rank *P*-value were computed. A meta-analysis was conducted to pool the above survival data of TWF1 using Revman 5.3 software.

1. ***Correlation of TWF1 and TMB/MSI***

The web of “http://sangerbox.com/Tool” (2) was used to analyze the potential correlation between *TWF1* expression and TMB (tumor mutational burden) or MSI (microsatellite instability) in TCGA tumors. Spearman’s rank correlation test was performed, and the P-value and partial correlation (cor) value were obtained.

**REFERENCES**

1. Kent WJ, Sugnet CW, Furey TS, Roskin KM, Pringle TH, Zahler AM, et al. The human genome browser at UCSC. *Genome Res*. (2002) 12:996-1006. doi: 10.1101/gr.229102

2. Bonneville R, Krook MA, Kautto EA, Miya J, Wing MR, Chen HZ, et al. Landscape of Microsatellite Instability Across 39 Cancer Types. *JCO Precis Oncol*. (2017) 2017. doi: 10.1200/PO.17.00073
